# Enhancing Native Plant Establishment in Mine Tailings under Drought Stress Conditions through the Application of Organo-Mineral Amendments and Microbial Inoculants

**DOI:** 10.3390/plants13060863

**Published:** 2024-03-17

**Authors:** Madline Atika, Benidire Leila, Sofia I. A. Pereira, Paula M. L. Castro, Boularbah Ali

**Affiliations:** 1 Laboratoire Bioressources et Sécurité Sanitaire des Aliments, Faculté des Sciences et Techniques, Université Cadi Ayyad, BP 549, Guéliz, Marrakech 40000, Morocco; l.benidire@uca.ma; 2 Ecole Supérieure de Technologie El Kelâa des Sraghna, Université Cadi Ayyad, Route de Béni Mellal Km 8 B.P 104, El Kelaa des Sraghna 43000, Morocco; 3 CBQF—Centro de Biotecnologia e Química Fina, Laboratório Associado, Escola Superior de Biotecnologia, Universidade Católica Portuguesa, Rua Diogo Botelho 1327, 4169-005 Porto, Portugal; sapereira@ucp.pt (S.I.A.P.); plcastro@ucp.pt (P.M.L.C.); 4 Center of Excellence for Soil and Africa Research in Africa, College of Agriculture and Environmental Sciences, Université Mohammed VI Polytechnique (UM6P), Benguerir 43150, Morocco

**Keywords:** contaminated areas, metals, phytoremediation, plant growth-promoting rhizobacteria, metallophytes

## Abstract

The implementation of phytoremediation strategies under arid and semiarid climates requires the use of appropriate plant species capable of withstanding multiple abiotic stresses. In this study, we assessed the combined effects of organo-mineral amendments and microbial inoculants on the chemical and biological properties of mine tailings, as well as on the growth of native plant species under drought stress conditions. Plants were cultivated in pots containing 1 kg of a mixture of mine tailings and topsoil (i.e., pre-mined superficial soil) in a 60:40 ratio, 6% marble sludge, and 10% sheep manure. Moreover, a consortium of four drought-resistant plant growth-promoting rhizobacteria (PGPR) was inoculated. Three irrigation levels were applied: well-watered, moderate water deficit, and severe water deficit, corresponding to 80%, 45%, and 30% of field capacity, respectively. The addition of topsoil and organo-mineral amendments to mine tailings significantly improved their chemical and biological properties, which were further enhanced by bacterial inoculation and plants’ establishment. Water stress negatively impacted enzymatic activities in amended tailings, resulting in a significant decrease in acid and alkaline phosphatases, urease, and dehydrogenase activities. Similar results were obtained for bacteria, fungi, and actinomycete abundance. PGPR inoculation positively influenced the availability of phosphorus, total nitrogen, and organic carbon, while it increased alkaline phosphatase, urease (by about 10%), and dehydrogenase activity (by 50%). The rhizosphere of *Peganum harmala* showed the highest enzymatic activity and number of culturable microorganisms, especially in inoculated treatments. Severe water deficit negatively affected plant growth, leading to a 40% reduction in the shoot biomass of both *Atriplex halimus* and *Pennisetum setaceum* compared to well-watered plants. *P. harmala* showed greater tolerance to water stress, evidenced by lower decreases observed in root and shoot length and dry weight compared to well-watered plants. The use of bioinoculants mitigated the negative effects of drought on *P. harmala* shoot biomass, resulting in an increase of up to 75% in the aerial biomass in plants exposed to severe water deficit. In conclusion, the results suggest that the combination of organo-mineral amendments, PGPR inoculation, and *P. harmala* represents a promising approach to enhance the phytoremediation of metal-polluted soils under semiarid conditions.

## 1. Introduction

The wind dispersal of toxic trace elements from mine tailings in arid and semiarid environments is a globally recognized concern which requires attention to minimize the risk of biomagnification of these elements into the food chain [1]. Conventional techniques used for the remediation of contaminated soils are not eco-friendly and are expensive and energy intensive [2,3]. The use of cost-effective remediation strategies involving the use of plants and the associated microbial communities to restore the ecosystem services of contaminated and/or degraded areas has emerged as a highly promising approach in recent decades [4,5].

Phytostabilization, a form of phytoremediation, employs plants and their associated microbes to effectively immobilize pollutants in belowground tissues and/or in the rhizosphere. The ultimate goal of this approach is to create a plant canopy that prevents eolian dispersion and water erosion through the immobilization of pollutants [4,6]. However, the successful implementation of phytoremediation strategies in arid and semiarid environments faces challenges due to unfavorable climatic conditions such as high salinity, extreme temperatures, and drought, which compromise plant growth and biomass production [7,8]. Drought is the main limiting factor for plant development in these regions due to low rainfall rates and high temperatures. Therefore, under limited soil moisture, plants show a significant reduction in photosynthesis, leading to a reduction in leaf expansion and, consequently, premature leaf senescence [9,10]. To overcome this problem, the use of plant species that are tolerant to the main pollutants and well adapted to the prevailing local climate conditions is crucial for a successful phytostabilization strategy [1,7]. Previous studies have reported several plant species colonizing mining areas within arid and semiarid regions that exhibit traits similar to metallophytes. They are considered non-hyperaccumulator species, but they have high metal tolerance alongside exceptional salt tolerance, as well as the capacity of withstanding severe droughts [11,12,13,14,15]. These native plants are often considered ideal candidates for phytostabilization, given their inherent traits and ability to tolerate harsh local conditions [12,16,17].

In addition to climatic factors, mine tailings are typically characterized by a poor structure, very acidic pH, high metal concentrations, and low nutrient content, which adversely impacts both vegetation cover and microbial communities [18,19]. The use of organic and inorganic amendments such as biochar, compost, animal wastes, and liming has been largely reported to decrease the availability of metals by increasing soil pH and physical adsorption, while improving soil physical, chemical, and biological properties [7,19]. In particular, organic amendments increase soil organic carbon, which interacts with clay minerals, forming stable aggregates and improving soil structure and macroporosity. Moreover, organic matter holds hygroscopic water on the surface of soil particles and capillary water in soil pores, thereby increasing soil water retention, infiltration, and storage [20,21,22]. On the other hand, the establishment of vegetation cover in mine tailings enhances the heterotrophic microbial community, which may, in turn, promote plant growth and contribute to metal immobilization [23,24]. Exploring these beneficial interactions is a valuable tool to improve the overall effectiveness of phytoremediation strategies in metal-contaminated areas. Several authors have reported the beneficial effects of inoculating plant growth-promoting rhizobacteria (PGPR) on plant establishment and development in metal-contaminated areas [6,8,23,25,26,27]. Microbial inoculants are known to decrease the availability of contaminants and enhance plant growth under stressed conditions through various mechanisms, including the production of indole-3-acetic acid (IAA) and siderophores, the solubilization of mineral phosphorus, and the activity of the enzyme 1-aminocyclopropane-1-carboxylate (ACC) deaminase [8,23,26]. Furthermore, the use of drought-tolerant strains confers advantages to the host plant against water deficiency, especially in arid and semiarid areas [28].

The present study aimed to evaluate the effects of microbial inoculants and organo-mineral amendments on the improvement to the chemical and microbiological properties of mine tailings. The ability of different native metal-tolerant plants to grow in amended mine tailings under moderate and severe drought stress conditions was also evaluated.

## 2. Results

### 2.1. Influence of Irrigation Regimes and Microbial Inoculants on Soil Chemical Properties

The addition of topsoil and organo-mineral amendments significantly improved mine tailings’ properties, since the pH increased (7.55), as well as the total nitrogen and organic carbon content, which stood at 0.32% and 3.11%, respectively (Table 1). We also observed a remarkable reduction in EC, although it remained slightly high (4.71 ± 0.04 mS cm^−1^). Post-harvest analyses showed that chemical properties varied across the three plant species and treatments tested (Table 1). When compared to the pre-plant values, the pH slightly decreased for all plant species. However, irrigation regimes and inoculation had a marginal influence on pH, despite the slight decrease observed in the rhizosphere of *P. harmala* plants with increasing water deficiency. A similar trend was observed for EC. In general, bacterial inoculation significantly increased the available concentrations of phosphorus (P Olsen) when compared to non-inoculated treatments, with higher increases being observed in the rhizosphere of *P. setaceum*. The cultivation of plants caused a remarkable increase in organic carbon content (≈1.3-fold) and in total nitrogen (≈1.5-fold) when compared to the pre-plant values. Interestingly, PGPR inoculation also enhanced total nitrogen and organic carbon content across all plants and irrigation treatments.

### 2.2. Influence of Irrigation Regimes and Microbial Inoculants on Soil Microbial Activity and Structure

**Figure 1:**
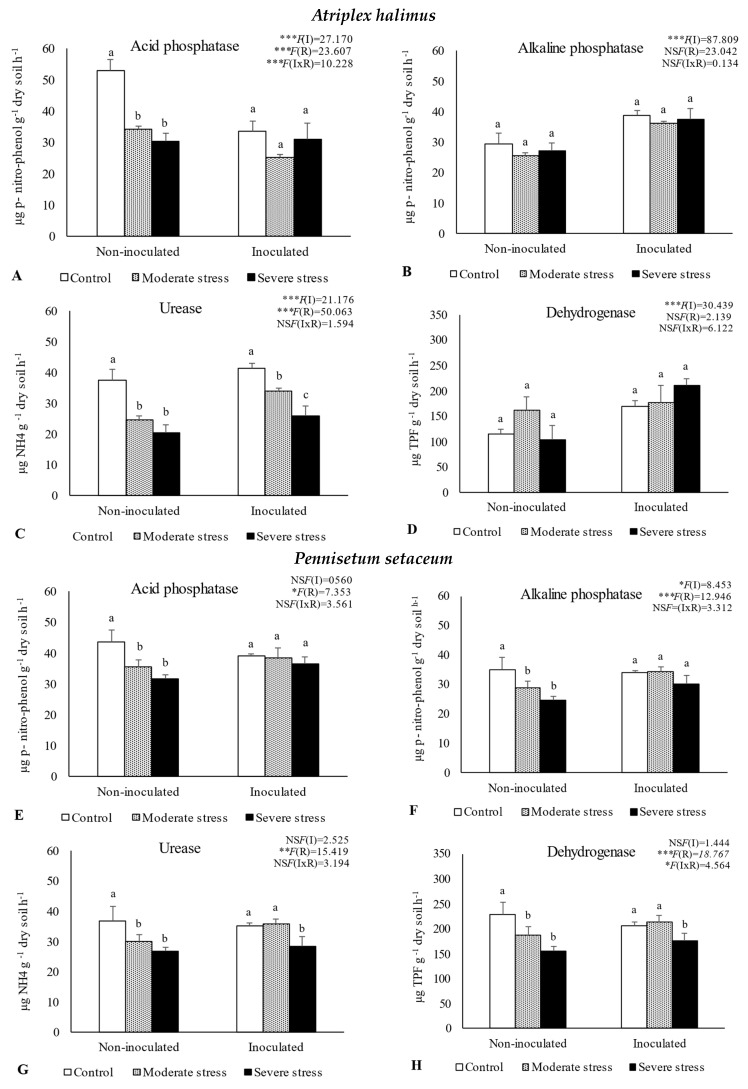

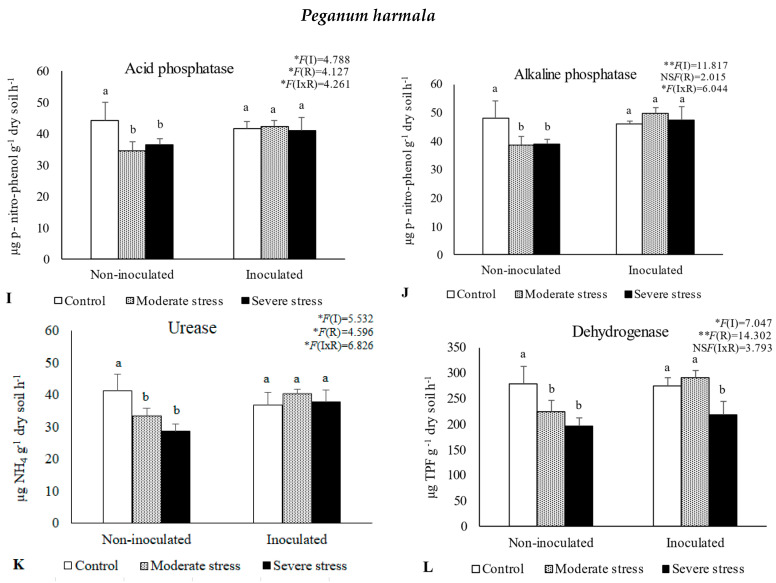
Activities of acid phosphatase (**A**,**E**,**I**), alkaline phosphatase (**B**,**F**,**J**), urease (**C**,**G**,**K**), and dehydrogenase (**D**,**H**,**L**) in the rhizosphere of different plant species: *Atriplex halimus*, *Pennisetum setaceum,* and *Peganum harmala*. Plants were grown under non−inoculated and inoculated conditions and under three water irrigation regimes: Control—watered at 80% of field capacity (FC); Moderate Stress—watered at 45% of FC; Severe Stress—watered at 30% of FC. Data are shown as means ± standard errors (n = 3). A two−way ANOVA was performed to determine the influence of microbial inoculation (I) and irrigation regimes (R) on enzymatic activity (acid phosphatase, alkaline phosphatase, urease, and dehydrogenase). The results are shown with the test statistic for each case (I: microbial inoculation; R: irrigation regimes; I x R: microbial inoculation x irrigation regimes) and as NS (non−significant) at the level *p* > 0.05; * significant at the level *p* < 0.05; ** *p* < 0.01; *** significant at the level *p* < 0.001, respectively. One−way ANOVA was performed to determine the influence of irrigation on enzymatic activity for each inoculation treatment at the end of the experiment. Means for the same inoculation treatment showing different letters are significantly different from each other (*p* < 0.05) according to Duncan’s test.

The activities of acid and alkaline phosphatases, dehydrogenase, and urease determined in the rhizosphere of the three plant species cultivated under different irrigation regimes and with and without inoculation at the end of the experiment are shown in Figure 1. The activity of acid phosphatase was reduced under moderate and severe water stress when compared with well-watered conditions. However, bacterial inoculation seemed to mitigate the negative impact of water stress on this enzyme, since no significant decreases were observed among the different irrigation regimes in inoculated soils. Similar results were observed for alkaline phosphatase and urease, where bacterial inoculation increased both enzymes’ activity by 10%, regardless of water stress conditions. The activity of dehydrogenase within the rhizosphere of *P. setaceum* and *P. harmala* plants experienced a decline under moderate and severe water stress conditions. Nonetheless, bacterial inoculation marginally enhanced the activity of this enzyme, with beneficial effects only observed in the rhizosphere of *P. harmala* under moderate water stress. Maximum values of alkaline phosphatase, urease, and dehydrogenase were observed in the rhizosphere of *P. harmala*, particularly when supplemented with the bacterial inoculum.

The number of viable bacteria, fungi, and actinomycetes changed remarkably across different treatments and plant species (Table 2). Notably, bacterial and actinomycete numbers were significantly higher in the rhizosphere of *P. harmala* in comparison to the rhizospheres of *A. halimus* and *P. setaceum*. Regarding fungi counts, no significant differences were observed among plant species. In general, increasing levels of drought stress led to a significant reduction in microbial counts. In the rhizosphere of *P. harmala*, water deficiency decreased the number of bacteria, fungi, and actinomycetes by 17%, 40%, and 30%, respectively, compared to control (well-watered conditions). Furthermore, across the three studied plant species, we observed a greater abundance of actinomycetes and bacteria in the rhizosphere of inoculated plants when compared to those that were non-inoculated. Similar results were obtained for fungi.

### 2.3. Influence of Irrigation Regimes and Microbial Inoculants on Plant Growth

**Figure 2:**
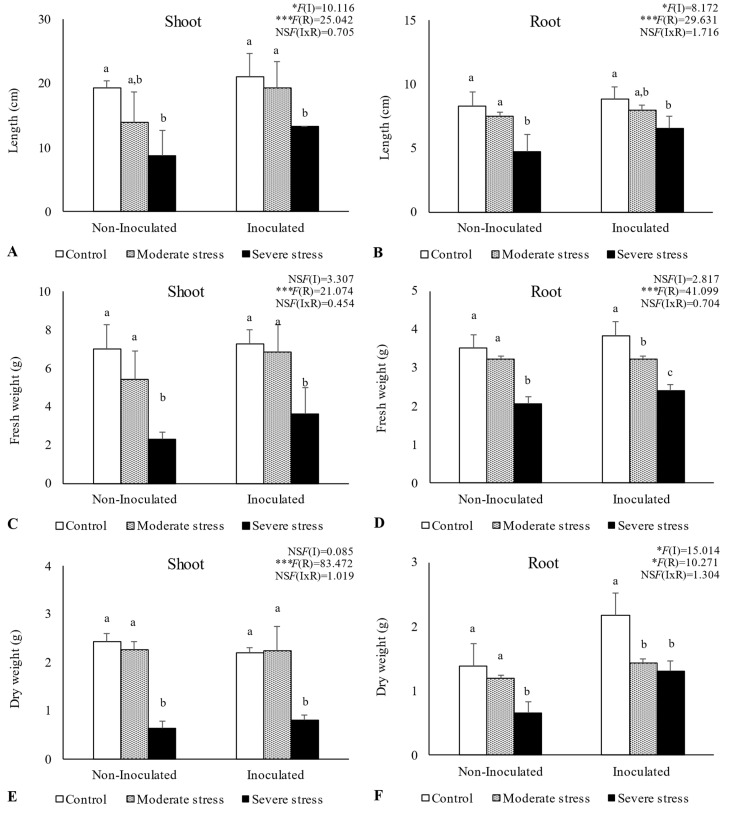
Shoot length (**A**), root length (**B**), shoot fresh biomass (**C**), root fresh biomass (**D**), shoot dry biomass (**E**), and root dry biomass (**F**) of *Atriplex halimus* growing under different water regimes (Control—watered at 80% of field capacity (FC); Moderate Stress—watered at 45% of FC; Severe Stress—watered at 30% of FC) and under non-inoculated and inoculated conditions. Data are shown as means ± standard errors (n = 3). A two−way ANOVA was performed to determine the influence of microbial inoculation (I) and irrigation regimes (R) on plant growth parameters (shoot length, root length, shoot fresh biomass, root fresh biomass, shoot dry biomass, and root dry biomass). The results are shown with the test statistic for each case (I: microbial inoculation; R: irrigation regimes; I x R: microbial inoculation x irrigation regimes) and as NS (non-significant) at the level *p* > 0.05; * significant at the level *p* < 0.05; *** significant at the level *p* < 0.001, respectively. One−way ANOVA was performed to determine the influence of irrigation regimes on plant growth parameters for each inoculation treatment at the end of the experiment. Means for the same inoculation treatment showing different letters are significantly different from each other (*p* < 0.05) according to Duncan’s test.

**Figure 3:**
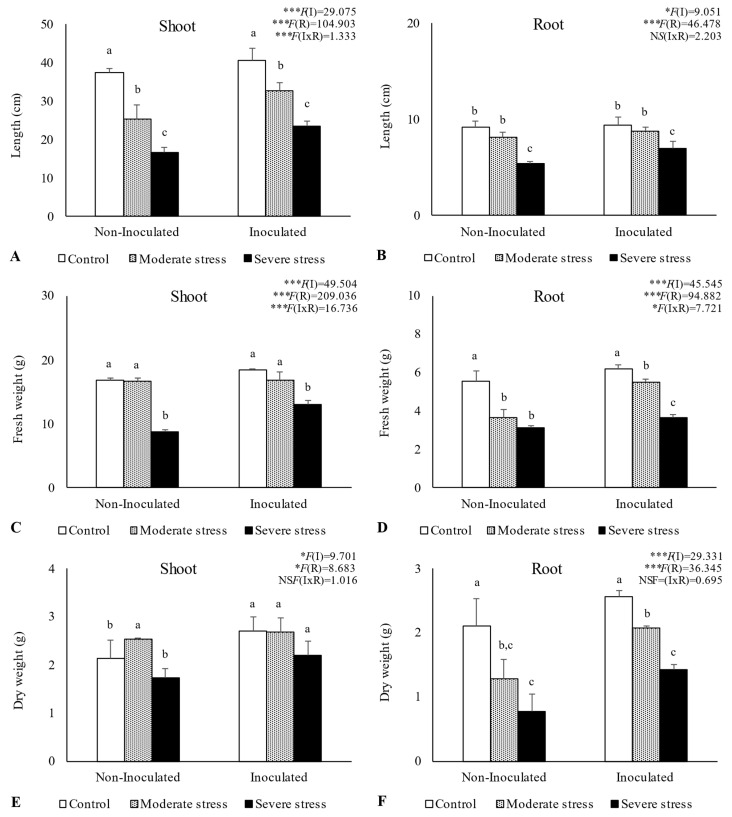
Shoot length (**A**), root length (**B**), shoot fresh biomass (**C**), root fresh biomass (**D**), shoot dry biomass (**E**), and root dry biomass (**F**) of *Pennisetum setaceum* growing under different water regimes (Control—watered at 80% of field capacity (FC); Moderate Stress—watered at 45% of FC; Severe Stress—watered at 30% of FC) and under non-inoculated and inoculated conditions. Data are shown as means ± standard errors (n = 3). A two-way ANOVA was performed to determine the influence of microbial inoculation (I) and irrigation regimes (R) on plant growth parameters (shoot length, root length, shoot fresh biomass, root fresh biomass, shoot dry biomass, and root dry biomass). The results are shown with the test statistic for each case (I: microbial inoculation; R: irrigation regimes; I x R: microbial inoculation x irrigation regimes) and as NS (non-significant) at the level *p* > 0.05; * significant at the level *p* < 0.05; *** significant at the level *p* < 0.001, respectively. One-way ANOVA was performed to determine the influence of irrigation regimes on plant growth parameters for each inoculation treatment at the end of the experiment. Means for the same inoculation treatment showing different letters are significantly different from each other (*p* < 0.05) according to Duncan’s test.

**Figure 4:**
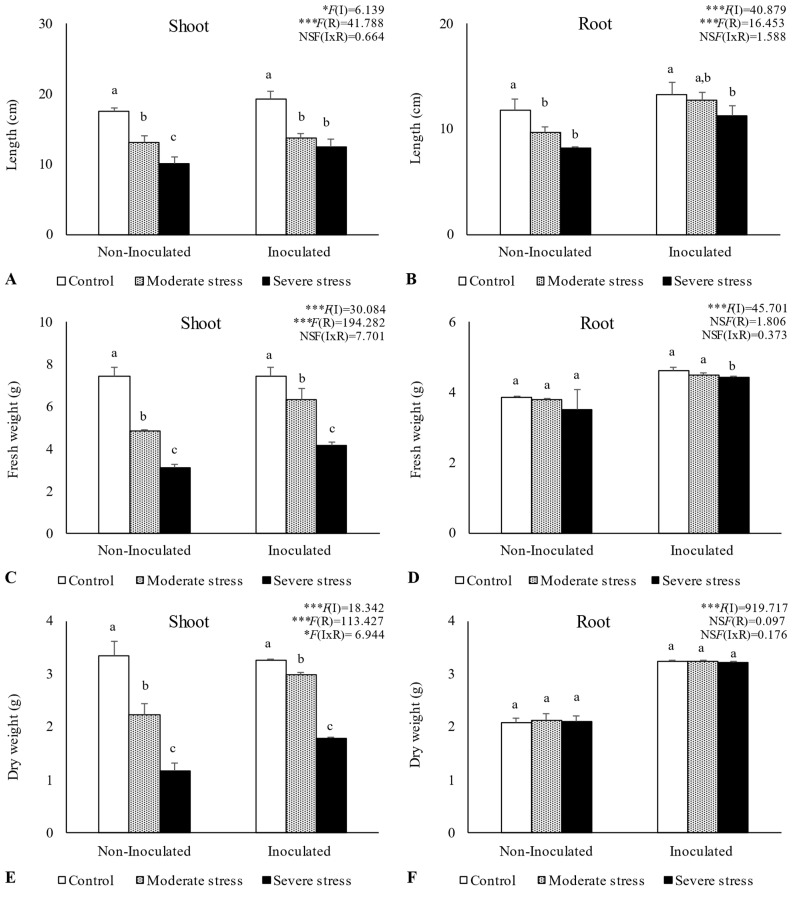
Shoot length (**A**), root length (**B**), shoot fresh biomass (**C**), root fresh biomass (**D**), shoot dry biomass (**E**), and root dry biomass (**F**) of *Peganum harmala* growing under different water regimes (Control—watered at 80% of field capacity (FC); Moderate Stress—watered at 45% of FC; Severe Stress—watered at 30% of FC) and under non-inoculated and inoculated conditions. Data are shown as means ± standard errors (n = 3). A two-way ANOVA was performed to determine the influence of microbial inoculation (I) and irrigation regimes (R) on plant growth parameters (shoot length, root length, shoot fresh biomass, root fresh biomass, shoot dry biomass, and root dry biomass). The results are shown with the test statistic for each case (I: microbial inoculation; R: irrigation regimes; I x R: microbial inoculation x irrigation regimes) and as NS (non-significant) at the level *p* > 0.05; * significant at the level *p* < 0.05; *** significant at the level *p* < 0.001, respectively. One-way ANOVA was performed to determine the influence of irrigation regimes on plant growth parameters for each inoculation treatment at the end of the experiment. Means for the same inoculation treatment showing different letters are significantly different from each other (*p* < 0.05) according to Duncan’s test.

Overall, water stress significantly affected plant growth, by reducing shoot and root length, as well as fresh and dry biomass (Figure 2, Figure 3 and Figure 4). For *A. halimus*, shoot and root length decreased by 52% and 50%, respectively, compared to well-watered plants under severe drought (Figure 2A,B). A similar trend was observed for *P. setaceum,* with a reduction of 47% and 66% for shoot and root length, respectively (Figure 3A,B). Nevertheless, *P. harmala* showed comparatively less sensitivity to water stress, as we observed a lower decrease in root and shoot length, 16% and 35%, respectively (Figure 4A,B). Shoot and root fresh weight were also significantly affected by water stress, with remarkable reductions being observed with increasing stress severity. Thus, severe drought stress caused a significant decrease in the root dry weight of *A. halimus* and *P. setaceum*, nearly 42% and 32%, respectively, while the root dry weight of *P. harmala* plants was not significantly affected (Figure 4F). However, moderate drought stress did not significantly affect the root and shoot dry weights of all plant species. Despite the substantial decrease in the shoots’ length and in dry and fresh weight with increasing drought stress intensity, plants did not visually exhibit symptoms of toxicity such as chlorosis.

Bacterial inoculation led to a slight increase in shoot and root length under well-watered conditions. However, the impact of PGPR inoculation was even more pronounced under water stress conditions. Indeed, under moderate and severe water stress, *A. halimus* and *P. setaceum* plants showed significant increases in shoot and root lengths upon PGPR inoculation (Figure 2A,B and Figure 3A,B). Higher increases were observed for *A. halimus* and *P. setaceum*, which corresponded to 35% and 39%, respectively. Likewise, the application of PGPR led to an increase in the dry weight of the roots of all three plants species. *P. harmala* showed the most significant enhancement, with an increase of 59% in roots and 56% in shoots, respectively, under severe stress conditions (Figure 4E,F).

## 3. Discussion

The addition of MBS and SM together with uncontaminated topsoil contributed to an increase in the nutrient content of mine tailings, enabling successful seed germination and subsequent plant establishment in mine tailings.

### 3.1. Improvement of Mine Tailings’ Chemical and Microbiological Properties by the Combined Use of Organo-Mineral Amendments and Bacterial Inoculants

At the end of the experiment, a decrease in pH was observed, which is aligned with previous research, as plants are known to cause the acidification of the rhizosphere due to the secretion of exudates by the roots, including protons, amino acids, and sugars [29,30,31]. These exudates may influence not only soil pH but also metal solubility and its speciation in the rhizosphere [32,33]. The decrease in pH can also be related to the significant build-up of CO_2_ in the rhizosphere resulting from the respiratory activities of both roots and microorganisms [29,34]. Further, numerous studies have reported that soil acidification is due to the release of organic acids during organic matter decomposition by microbes [29].

Soil organic matter is essential to soil function and quality. Our results revealed a significant increase in soil organic carbon and total nitrogen following plant growth and bacterial inoculation. This finding may be related to the slough of root cells, the presence of delicate root hairs, and the release of organic compounds by the roots. These processes create a unique environment that promotes microbial activity, which, in turn, contributes to the mobilization of nitrogen and phosphorus within the soil [29,30,31,35]. Furthermore, previous studies have emphasized the crucial role of free-living N_2_-fixing bacteria in both nitrogen fixation and the enhancement of nutrient availability to plants [23,36,37]. The inoculated consortium comprises strains with the capability to produce IAA and ammonia, which may also have contributed to increasing the total nitrogen content in inoculated rhizospheres [38]. Similarly, plant establishment and PGPR inoculation enhanced the concentration of available phosphorus. These results may be attributed to the ability of inoculated strains to convert insoluble P present in MBS and SM into soluble forms, such as H_2_PO_4_^−^, by releasing organic acids and phosphatases. Indeed, several authors highlighted the notable role of PGPR in the solubilization of inorganic phosphate and the mineralization of organic phosphate [39,40,41,42,43].

Soil microbial communities and their associated enzymatic activity are considered fundamental to soil biochemical cycles. Indeed, soil enzymes mediate many nutrient transformations and organic matter decomposition processes that are closely related to soil fertility. In this study, the results clearly demonstrated the negative impact of water stress on enzymatic activity, through the decrease in the activity of enzyme acid and alkaline phosphatase, urease, and dehydrogenase. The impact of water deficiency on soil enzymatic activities has been well documented [44,45,46,47,48,49]. Microorganisms play a crucial role in soil fertility by producing and releasing enzymes to obtain nutrients, thereby highlighting the importance of soil enzymes as indicators of soil microbial viability [50,51]. The decrease in soil moisture due to drought can restrict microbial physiological processes and the availability of substrates essential for enzyme production, leading to a decline in their activity [47,49].

**Figure 5:**
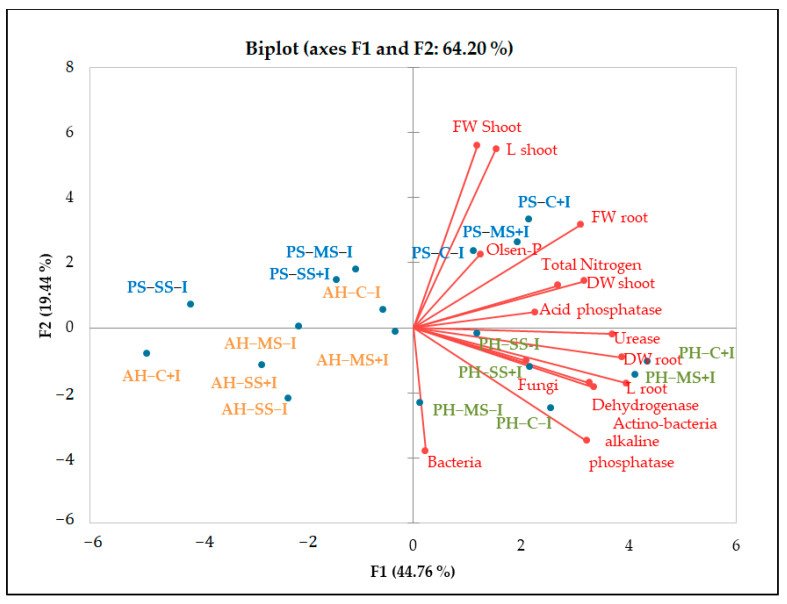
PCA diagram of correlations between chemical and biological parameters of non-inoculated and inoculated plants under different water regimes. The red lines represent the vectors of each parameter. The correlation is described by the cosine of the angle between vectors. The smaller the angle, the higher the correlation between parameters. Uncorrelated parameters are orthogonal to each other. The size of the vectors relative to the designated PC serves as an indicator of a parameter’s significance for that component. PH: *Peganum harmala*, AH: *Atriplex halimus*, PS: *Penisetum setaceum;* C: control (well−watered), MS: moderated stress, SS: severe stress, +I: inoculated, −I: non inoculated, FW: fresh weight, DW: dry weight.

PCA analysis underlined the positive relationship between soil enzymatic activity and available phosphorus, organic carbon, and total nitrogen (Figure 5. The results also revealed significant variations in enzymatic activity across plant species. Notably, higher values of alkaline phosphatase, urease, and dehydrogenase were observed in the rhizosphere of *P. harmala*. Likewise, a higher abundance of bacteria and actinomycetes was observed in *P. harmala’s* rhizosphere than in the rhizospheres of *A. halimus* and *P. setaceum*. These differences can be attributed to the distinctive root morphology of each plant species, coupled with variations in the quantity and quality of their respective rhizodeposits. All together, these factors are able to significantly influence the composition and functioning of the rhizosphere microbiome, potentially leading to an increase in soil enzymatic activities. Our results are consistent with previous studies conducted by [50] and [30]. These studies corroborate the role of plant species in shaping soil microbial biomass and enzymatic activity by providing substrates that stimulate enzyme synthesis. In line with this, [51] reported that successive plantations of *Eucalyptus urophylla* resulted in a decrease in soil bacterial community diversity, microbial biomass, and enzyme activity. Conversely, [52] reported that growing *Trifolium repens* in metal-contaminated soil mitigated the decline in enzyme activity caused by contamination. This was achieved through an increase in the soil’s organic matter content resulting from the release of root exudates and the accumulation of litter.

In this study, dehydrogenase activity was significantly increased through inoculation with PGPR, which may be related to the production of enzymes by the bacterial strains present in the inoculum or through their interactions with native microorganisms [42,51,53]. Furthermore, actinomycetes and bacterial counts were higher in the rhizosphere of inoculated plants, suggesting that the selected bacteria possess the ability to foster native microbial communities, a characteristic that could also explain their positive impact on soil enzymatic activity. Furthermore, bacterial inoculum seems to enhance nutrient content in the rhizosphere, improving the plant–root system and supporting microbe diversity and activity [42,50,53]. PGPR are able to produce exopolysaccharides, which are extracellular polymeric substances that enhance water retention and thus regulate the diffusion of organic carbon sources, facilitating nutrient and water uptake by root cells [54,55], which is crucial to cope with metal exposure and water deficiency. This further demonstrates the multifaceted ways in which PGPR may enhance soil conditions and ultimately influence enzymatic activities.

### 3.2. Plant Growth in Amended Mine Tailings under Water Stress Conditions

Our results highlight the potential of the three plant species to be cultivated in amended mine tailings. The improvements in soil fertility attributed to the incorporation of marble sludge and sheep manure had profound effects on plant growth. The pH adjustments provided by marble sludge and the nutrient enrichment provided by sheep manure acted synergistically to enhance the growth of both rhizospheric microorganisms and plants.

Plant growth was negatively influenced by drought stress, with *P. setaceum* being the most affected species. In contrast, *P. harmala* and *A. halimus* demonstrated a higher resilience to drought stress, suggesting evolutionary adaptations to endure harsh environmental conditions. Additionally, further analysis (Figure 3 and Figure 5) showed that *P. harmala* exhibited higher shoot biomass production and root elongation in comparison to *A. halimus*. Ref. [56] also reported reductions in the root and shoot biomass of *Atriplex* sp. Under water stress conditions. Indeed, drought stress is known to influence key aspects of plant growth such as root diameter, root volume, and the process of root differentiation [57]. Moreover, it can also negatively impact the balance of endogenous hormones in roots, leading to a decrease in IAA and a simultaneous increase in abscisic acid content [57]. The complex dynamics between soil moisture and metal toxicity add another layer of complexity to this phenomenon. Indeed, it has been reported that a decrease in water availability may increase metal toxicity in soil solution, which subsequently hinders root growth [58,59].

In this study, *P. harmala* arose as a suitable plant to withstand the challenges posed by both drought and metal toxicity. Despite the supplementation of mine tailings with topsoil and organo-mineral amendments, the Cu concentration remained significantly high, surpassing the limits allowed for industrial land use specified in the Canadian Soil Quality Guidelines. These findings are in line with previous works that show the capacity of *P. harmala* to thrive in the presence of a wide range of metals while developing a resilient and deeply rooted system [60]. This adaptability might be attributed to its ability to release high- and low-molecular-weight root exudates, which shield the root apex from metal toxicity during severe environmental stresses [61,62,63]. These exudates, coupled with modifications in cell wall and plasma membrane properties, provide mechanical strength to the root system, further enhancing its ability to withstand harsh abiotic stressors. Furthermore, the preservation of root elongation, through osmotic adjustment and abscisic acid accumulation, has also been highlighted as a key factor for sustaining root growth under adverse conditions [64]. These strategies corroborate the potential of *P. harmala* plants to cope with environmental challenges, demonstrating their adaptability and resilience.

The outcomes of the present study showed that bacterial inoculation had a pronounced and positive impact on plant growth, which was particularly evident under water stress conditions. The improvement in plant growth by PGPR inoculation in metal-contaminated soils and under drought stress conditions could be attributed to their capacity to produce siderophores and phytohormones (e.g., IAA) and to solubilize inorganic phosphate, in addition to the activity of the enzyme ACC-deaminase. Moreover, the rhizobacteria used as inoculum showed a high ability to produce exopolysaccharides and ammonia under different abiotic stresses, including drought, metal, and salt. Exopolysaccharide-producing bacteria are acknowledged for their role in preserving soil moisture and enhancing plant growth, even in arid conditions. Furthermore, ammonia produced by bacteria acts as an extra nitrogen source for the host plant, leading to an increase in overall plant biomass [65,66,67]. Moreover, the synthesis of IAA by plant-associated rhizobacteria appears to be an additional factor contributing to plant growth in metal-polluted soils, through the stimulation of plant root elongation, which enhances plant nutrient uptake [8,68]. Moreover, the concurrent application of sheep manure and PGPR inoculation plays a pivotal role in stimulating soil microbial activity, thereby enriching plant nutrition. The introduction of organic amendments appears to be a carbon source as well as an energy reservoir for the inoculated bacteria, fostering beneficial microbial activity and consequently enhancing their functional traits that contribute to improved plant nutrition.

## 4. Materials and Methods

### 4.1. Plant Species

Three plant species were used: *Atriplex halimus* (Chenopodiaceae), *Pennisetum setaceum* (Poaceae), and *Peganum harmala* (Zygophyllaceae). These species were selected as they are native perennials, thriving within the geographical area of the study. In addition, they show high tolerance to metallic stress and are well adapted to arid conditions, especially to hot summers and low annual rainfall. Moreover, members of the Chenopodiaceae family, specifically *Atriplex* spp., are known to exhibit exceptional tolerance to both salinity and drought, while *P. setaceum* is a salt-resistant perennial herb that can provide an extensive and dense canopy cover, coupled with a well-established deeper root network, effectively preventing erosion [69]. On the other hand, *P. harmala* is a perennial herb that thrives naturally in degraded and metalliferous lands within arid and semiarid regions. Notably, its root system can reach impressive depth, ranging from 5 to 6 m. Given these remarkable attributes, these plants species are highly effective in stabilizing contaminants in soils and may act as pioneers for the revegetation of these areas.

The seeds of *P. harmala* and *P. setaceum* were collected in the vicinity of Kettara mine tailings, while plants of *A. halimus* were obtained through transplantation to ensure their establishment and growth for experimental purposes.

### 4.2. PGPR Consortium, Seed Sterilization, and Seedling Growth

In this assay, a consortium consisting of four bacterial strains, namely *Mesorhizobium tamadayense* BKM04, *Enterobacter xiangfangensis* BKM30, *Pseudomonas azotifigens* BKM07, and *Streptomyces caelestis* BKM05, was used. These strains were chosen based on their proven ability to withstand environmental stressors, including drought, salt stress, and high levels of metals, as well as their capacity to promote plant growth [27,70]. To prepare the inoculum, single colonies of each strain were transferred from tryptic soy agar (TSA) plates to a nutrient broth and incubated with agitation (200 rpm) at 28 °C for 48 h. Subsequently, the cultures were centrifuged at 10,000 rpm for 10 min, and the pellet was washed twice with a sterile saline solution (0.9% (*w*/*v*) NaCl). Bacterial cells were then resuspended in 1% (*w*/*v*) of methylcellulose solution prepared in 10 mM of MgSO4. Equal numbers of cells of each rhizobacteria were mixed to form the inoculum (≈2 × 10^8^ colony forming units (CFU) mL^−1^).

Seeds of *P. harmala* and *P. setaceum* were sterilized using 70% ethanol followed by 3% sodium hypochlorite for 5 min. Subsequently, seeds were washed several times with deionized sterilized water. Sterilized seeds were then soaked in the inoculum for 30 min. For *A. halimus*, young shoots were collected from plants growing in their natural habitat. The collected plant material was first thoroughly washed in running tap water for 15 min to remove any dust and other particles. Explants of 5–7 cm, containing the nodes and axillary bud, were excised from the shoots. These explants were subjected to surface sterilization with 70% ethanol for 1 min and subsequently rinsed several times with sterile distilled water. Finally, they were carefully placed in plastic cups containing a mixture of agriculture soil and peat and kept under greenhouse conditions for 2 months. During this period, the plants were irrigated 1–2 times per week until they were ready for their final transplantation into pots for the experiment. Rooted plants were then inoculated with 10 mL of bacterial inoculum after transplantation to the pots.

### 4.3. Sampling and Characterization of Mine Tailings, Topsoil, and Organo-Mineral Amendments

Mine tailings and topsoil (upper soil layer removed prior to rock mine excavation) were collected from an abandoned pyrrhotite mine (Kettara), located 30 km of northwest of Marrakech (Morocco). Mine tailings are highly contaminated with various metals, posing serious environmental problems [12,13,70,71]. Topsoil and organic (sheep manure) and inorganic (crushed marble sludge) amendments were used to reduce metal toxicity and improve the chemical properties of tailings. Sheep manure (SM) was collected from a sheep farm in Ourika located 32 km south of Marrakech (Morocco), while crushed marble sludge (MBS) was collected from the industrial zone of Marrakech (Sidi Ghanem). The physicochemical properties of mine tailings, topsoil, and amendments are shown in Table 3. Mine tailings and topsoil were sieved (<2 mm) and mixed in a ratio of 40% of topsoil and 60% of tailings. Then, 6% of MBS and 10% of SM were added. The selection of these ratios was based on a preliminary experiment conducted in our laboratory. Various mixtures of mine tailings and topsoil were supplemented with different proportions of MBS and SM. Among these, the treatment that yielded the most notable results in mitigating mine tailings’ toxicity and enhancing nutrient levels, microbial activity, and seed germination and growth comprised 40% topsoil, 60% mine tailings, 6% MBS, and 10% SM.

### 4.4. Greenhouse Pot Experiment

Pots containing 1 kg of the prepared mixture (Section 4.3) were irrigated with deionized water to reach 70% of field capacity. Then, they were transferred to the greenhouse and left to stabilize for 30 days. Following this period, 0.3 g of *P. harmala* and 0.5 g of P. setaceum non-inoculated and inoculated seeds were sown in pots. Three rooted plants of *A. halimus* of similar size (2 months old) were carefully transplanted to the pots containing the mixture. Subsequently, in the treatments with inoculation, 10 mL of the bacterial consortium was added.

All pots were watered to reach 70% of field capacity with distilled water. After emergence (≈30 days after sowing), the *P. harmala* plants were selectively thinned to 3, while *A. halimus* were thinned to one. A re-inoculation was performed four weeks after the emergence of *P. harmala* and *P. setaceum* seedlings, as well as after the transplantation of *A. halimus* cuttings. To simulate real environmental conditions, 3 irrigation regimes corresponding to 3 levels of water deficit were applied one week after reinoculation for both microbial treatments (non-inoculated and inoculated): Control—plants watered at 80% of field capacity (FC); Moderate Stress—plants watered at 45% of FC; and Severe Stress—plants watered at 30% of FC.

The experiment was conducted in semi-controlled greenhouse conditions under natural light, with the temperature adjusted to 30 °C. Each treatment was replicated 3 times.

After 4 months, soil and plants were harvested and analyzed. Soil samples were sieved through a 2 mm mesh to eliminate particulate matter and plant debris. The samples were then divided into 2 fractions: the first fraction was stored at 4 °C for biological analysis, while the second one was air-dried for subsequent physicochemical analysis.

#### 4.4.1. Soil Physicochemical Analysis

Soil pH and electrical conductivity (EC) were determined in a soil–distilled water suspension with a ratio of 1:2.5 (*w*/*v*) and 1:5 (*w*/*v*), respectively. The total nitrogen amount was determined using the Kjeldahl method based on soil mineralization with sulfuric acid at high temperature. The molybdenum blue method was employed to determine the available phosphorus [72], while organic carbon was estimated using the oxidation of organic matter by potassium dichromate as outlined by [73].

#### 4.4.2. Soil Enzymatic Activity

Acid and alkaline phosphomonoesterase activities were measured by incubating soil with a substrate containing a p-nitrophenyl group according to [74]. Briefly, a mixture of 1 g of soil, 4 mL of modified universal buffer, 0.25 mL of toluene, and 1 mL of 15 mM p-nitrophenyl phosphate (*p*NPP) was incubated for 1 h at 37 °C. After stopping the reaction with 1 mL of calcium chloride (0.5 M) and 4 mL of sodium hydroxide (0.5 M), the color intensity was measured at 400 nm to quantify phosphatase activity as μg of p-nitrophenol released per g of dry soil per hour (µg p-nitrophenol g^−1^ h^−1^). Dehydrogenase activity was quantified following the method in [75], based on the reduction of 2,3,5-triphenyltetrazolium chloride (TTC) to 1,3,5-triphenyltetrazolium formazan (TPF). Each soil sample (5 g) was incubated with 5 mL of 0.8% TTC for 24 h in the dark at 37 °C. The formed TPF was subsequently extracted using acetone and analyzed spectrophotometrically at 546 nm. Dehydrogenase activity was expressed as µg TPF released per gram of dry soil per hour (µg^−1^ dry soil h^−1^). Urease activity was evaluated using the method in [76], involving the determination of the ammonium concentration resulting from soil and urea incubation. Three replicates were carried out for all assays.

#### 4.4.3. Enumeration of Culturable Microorganisms

The plate count technique was used to quantify the viable microbial population (CFU g^−1^ dry soil) in each sample. Serial dilutions were prepared by suspending 10 g of soil in 90 mL sterile saline solution (0.9% NaCl) and shaken for 30 min. After 15 min standing, serial dilutions (from 10^−1^ to 10^−9^) were prepared. From each dilution, 100 μL was spread onto TSA medium supplemented with 50 mg L^−1^ of actidione and incubated at 28 °C for 48 h for bacteria. Fungi were cultivated and incubated at 20 °C for 7 days on Potato Dextrose Agar (PDA) medium supplemented with Rose Bengal, while actinomycetes were grown on International Streptomyces Project 2 (ISP2).

#### 4.4.4. Plant Biometric Parameters

After four months of the experiment, plants were harvested, thoroughly washed with tap water to remove any attached particles, and then rinsed with deionized water. Shoot height was measured, as well as the dry biomass of shoots and roots after drying the samples at 70 °C for 48 h.

#### 4.4.5. Statistical Analysis

A two-way ANOVA was performed to determine the influence of microbial inoculation (I) and irrigation regimes (R) on different parameters with the SPSS program (IBM, Armonk, NY, USA, version 25.0). One-way ANOVA was carried out to determine the influence of the irrigation regimes on the different parameters for each inoculation treatment at the end of the experiment. Student’s *t*-test was used to compare pre-plant and post-plant soil parameters. A statistical principal component analysis (PCA) based on Pearson’s correlation matrix was performed with XLSTAT to identify possible correlations between the biological and physicochemical parameters, taking into account the microbial inoculation and irrigation regimes for each plant species.

## 5. Conclusions

The present study highlights the beneficial effects of organo-mineral amendments and topsoil on the growth of *A. halimus; P. harmala,* and *P. setaceum* in mine tailings by improving their chemical and microbiological properties. Significant changes in pH, organic carbon, total nitrogen, and available phosphorus were observed, resulting from both plant growth and bacterial inoculation.

Microbial community abundance and enzymatic activities were different across the rhizospheres of the three plant species. Notably, the rhizosphere of *P. harmala* exhibited a higher number of bacteria and actinomycetes, as well as increased enzymatic activities compared to those found in the rhizospheres of *A. halimus* and *P*. *setaceum*. This suggests that root morphology and rhizodeposit quantity and quality play a pivotal role in shaping the composition and activity of the rhizosphere microbiome, subsequently influencing soil enzymatic activities.

Plant growth was significantly influenced by water stress, with decreases in shoot and root lengths, as well as in fresh and dry weights. *P. harmala* exhibited greater resilience to water stress compared to the other plant species. Furthermore, PGPR inoculation exerted a positive influence on plant growth, particularly under water-stressed conditions, driving heightened shoot and root lengths, alongside augmented dry weights.

The present study emphasizes the use of native plant species, like *P. harmala*, for reclaiming and restoring soil health in challenging environmental settings like metal-contaminated mine tailings in arid and semiarid regions. The successful integration of organo-mineral amendments and microbial inoculants further strengthens these efforts, offering a sustainable way to enhance soil quality and foster plant growth even under stressful conditions.

## Figures and Tables

**Table 1:** Physicochemical properties of amended mine tailings before plant establishment (pre-plant) and at the end of the experiment for different plant species: *Atriplex halimus*, *Penisetum setaceum,* and *Peganum harmala*. Plants were grown under non-inoculated and inoculated conditions and under three water irrigation regimes: Control—watered at 80% of field capacity (FC); Moderate Stress—watered at 45% of FC; Severe Stress—watered at 30% of FC.

			pH	EC (mS cm^−1^)	P Olsen (mg kg^−1^)	TN (%)	OC (%)
	Pre-plant		7.55 ± 0.20	4.71 ± 0.04	42.84 ± 0.61	0.32 ± 0.01	3.11 ± 0.05
*Atriplex halimus*	Non inoculated	Control	6.72 ± 0.04 ^a,^**	4.35 ± 0.02 ^b,^**	40.86 ± 3.63 ^b,NS^	0.51 ± 0.02 ^a,^**	4.70 ± 0.02 ^a,^**
		Moderate stress	6.62 ± 0.05 ^b,^**	4.34 ± 0.01 ^b,^**	40.17 ± 5.35 ^b,NS^	0.42 ± 0.03 ^b,^**	4.68 ± 0.11 ^a,^**
		Severe stress	6.75 ± 0.03 ^a,^**	4.42 ± 0.02 ^a,^**	52.68 ± 3.06 ^a,^**	0.38 ± 0.04 ^b,NS^	3.68 ± 0.09 ^b,^**
	Inoculated	Control	6.57 ± 0.02 ^a,^**	4.21 ± 0.04 ^b,^**	51.30 ± 5.31 ^a,^**	0.54 ± 0.01 ^a,^**	4.71 ± 0.04 ^a,^**
		Moderate stress	6.54 ± 0.05 ^a,^**	4.32 ± 0.05 ^a,^**	55.30 ± 5.85 ^a,^**	0.53 ± 0.04 ^a,^**	4.37 ± 0.58 ^a,^**
		Severe stress	6.51 ± 0.02 ^a,^**	3.90 ± 0.04 ^c,^**	55.31 ± 6.09 ^a,^**	0.38 ± 0.43 ^b,^**	4.68 ± 0.11 ^a,^**
		Two-Way ANOVA	*** *F*(I) = 72.532	*** *F*(I) = 180.549	** *F*(I) = 15.793	** *F*(I) = 7.506	NS *F*(I) = 0.015
			* *F*(R) = 5.277	*** *F*(R) *=* 36.093	* *F*(R) = 4.152	*** *F*(R) = 25.434	* *F*(R) = 10.111
			* *F*(IxR) *=* 6.004	* ** *F*(IxR) = 78.615	NS *F*(IxR) = 2.370	* *F*(IxR) = 3.945	NS *F*(IxR) = 1.393
*Penisetum setaceum*	Non inoculated	Control	6.66 ± 0.05 ^a,^**	4.21 ± 0.04 ^b,^**	47.97 ± 1.35 ^a,NS^	0.42 ± 0.02 ^a,^**	3.64 ± 0.02 ^a,^**
		Moderate stress	6.72 ± 0.03 ^a,^**	4.32 ± 0.05 ^a,^**	48.63 ± 0.15 ^a^	0.45 ± 0.07 ^a,^**	3.60 ± 0.03 ^a,^**
		Severe stress	6.72 ± 0.04 ^a,^**	3.90 ± 0.04 ^c,^**	45.31 ± 2.89 ^a,NS^	0.34 ± 0.04 ^b,^**	3.71 ± 0.04 ^a,^**
	Inoculated	Control	7.25 ± 0.01 ^a,^**	4.32 ± 0.02 ^b,^**	60.86 ± 4.38 ^a,^**	0.50 ± 0.07 ^bc,^**	4.20 ± 0.02 ^b,^**
		Moderate stress	6.51 ± 0.05 ^b,^**	4.34 ± 0.04 ^b,^**	63.51 ± 1.35 ^a,^**	0.54 ± 0.02 ^b,^**	4.20 ± 0.02 ^b,^**
		Severe stress	6.46 ± 0.30 ^b,^**	4.42 ± 0.02 ^a,^**	41.68 ± 3.50 ^b,NS^	0.56 ± 0.03 ^ab,^**	4.36 ± 0.03 ^a,^**
		Two-Way ANOVA	NS *F*(I) = 0.379	*** *F*(I) = 180.549	*** *F*(I) = 41.102	*** *F*(I) = 85.32	*** *F*(I) = 611.163
			*** *F*(R) = 14.706	** *F*(R) = 36.093	*** *F*(R) = 39.541	* *F*(R) = 4.316	* *F*(R) = 10.866
			*** *F*(IxR) = 19.82	*** *F*(IxR) = 78.61	*** *F*(IxR) = 21.87	** *F*(IxR) = 11.15	NS*F*(IxR) = 0.89
*Peganum harmala*	Non inoculated	Control	7.12 ± 0.02 ^a,^**	3.67 ± 0.02 ^c,^**	37.42 ± 2.50 ^a,NS^	0.42 ± 0.04 ^a,^**	3.81 ± 0.01 ^a,^**
		Moderate stress	6.44 ± 0.02 ^b,^**	4.35 ± 0.04 ^b,^**	40.29 ± 1.87 ^a,NS^	0.43 ± 0.01 ^a,^**	3.77 ± 0.02 ^a,^**
		Severe stress	6.35 ± 0.03 ^c,^**	4.46 ± 0.02 ^a,^**	38.21 ± 1.56 ^a,NS^	0.45 ± 0.03 ^a,^**	3.65 ± 0.04 ^b,^**
	Inoculated	Control	7.10 ± 0.02 ^a,^**	4.23 ± 0.01 ^a,^**	54.15 ± 1.78 ^c,^**	0.53 ± 0.07 ^a,^**	4.67 ± 0.05 ^a,^**
		Moderate stress	6.31 ± 0.02 ^b,^**	4.23 ± 0.03 ^a,^**	57.42 ± 0.94 ^b,^**	0.53 ± 0.08 ^a,^**	4.67 ± 0.01 ^a,^**
		Severe stress	6.32 ± 0.01 ^b,^**	3.87 ± 0.07 ^a,^**	60.29 ± 1.87 ^a,^**	0.51 ± 0.02 ^a,^**	4.43 ± 0.02 ^b,^**
		Two-Way ANOVA	*** *F*(I) = 119.102	* *F*(I) = 6.258	*** *F*(I) = 472.278	*** *F*(I) = 152.581	*** *F*(I) = 1293.901
			*** *F*(R) = 2258.193	*** *F(R*) = 110.180	* *F*(R) = 6.516	NS*F*(R) = 0.070	*** *F*(R) = 31.168
			NS*F*(IxR) = 2.261	*** *F*(IxR) = 309.705	* *F*(IxR) = 4.021	* *F*(IxR) = 4.395	NS*F*(IxR) = 2.806

Data are shown as means *±* standard errors (n = 3). A two-way ANOVA was performed to determine the influence of microbial inoculation (I) and irrigation regimes (R) on physicochemical parameters (pH; EC: electric conductivity; P Olsen: available P; TN: total nitrogen; OC: organic carbon content). The results are shown with the test statistic for each case (I: microbial inoculation; R: irrigation regimes; I x R: microbial inoculation x irrigation regimes) and as NS (non-significant) at the level *p* > 0.05; * significant at the level *p* < 0.05; ** *p* < 0.01; *** significant at the level *p* < 0.001, respectively. One-way ANOVA was performed to determine the influence of irrigation on pH, EC, P Olsen, TN, and TOC for each inoculation treatment at the end of the experiment. Means for the same inoculation treatment showing different letters are significantly different from each other (*p* < 0.05) according to Duncan’s test. Asterisks show significant differences between pre-plant and post-plant soil parameters according to Student’s *t*-test. NS: non-significant at the level *p* > 0.05; ** significant at the level *p* < 0.01.

**Table 2:** Total numbers of bacteria, fungi, and actinomycetes in the rhizosphere of different plant species: *Atriplex halimus*, *Penisetum setaceum,* and *Peganum harmala* at the end of the experiment. Plants were grown under non-inoculated and inoculated conditions and under three water irrigation regimes: Control—watered at 80% of field capacity (FC); Moderate Stress—watered at 45% of FC; Severe Stress—watered at 30% of FC.

			Bacteria (×10^7^ CFU g^−1^ dry soil)	Fungi (×10^4^ CFU g^−1^ dry soil)	Actinobacteria (×10^3^ CFU g^−1^ dry soil)
*Atriplex halimus*	Non inoculated	Control	10.79 ± 0.01 ^a^	2.33 ± 0.51 ^b^	28.72 ± 0.01 ^a^
		Moderate stress	10.95 ± 0.01 ^a^	5.00 ± 0.60 ^a^	24.05 ± 0.02 ^b^
		Severe stress	9.053 ± 0.02 ^b^	4.53 ± 0.11 ^a^	20.08 ± 0.04 ^c^
	Inoculated	Control	11.32 ± 0.01 ^a^	5.40 ± 0.30 ^a^	34.72 ± 0.01 ^a^
		Moderate stress	11.34 ± 0.03 ^a^	4.93 ± 0.57 ^a^	33.33 ± 0.03 ^a^
		Severe stress	10.33 ± 0.02 ^b^	3.20 ± 0.40 ^b^	22.72 ± 0.02 ^b^
		Two-Way ANOVA	*** *F*(I) = 105.585	* *F*(I) = 7.440	*** *F*(I) = 33.136
			*** *F*(R) = 182.036	*** *F*(R) = 12.964	*** *F*(R) = 34.682
			*** *F*(IxR) = 16.730	*** *F*(IxR) = 41.226	NS*F*(IxR) = 3.409
*Penisetum setaceum*	Non inoculated	Control	11.48 ± 0.48 ^a^	3.47 ± 0.23 ^a^	22.05 ± 0.20 ^a^
		Moderate stress	10.68 ± 0.40 ^b^	2.40 ± 0.01 ^b^	25.34 ± 0.41 ^b^
		Severe stress	9.413 ± 0.12 ^c^	1.80 ± 0.20 ^c^	14.72 ± 0.20 ^c^
	Inoculated	Control	12.63 ± 0.18 ^a^	4.13 ± 0.46 ^a^	25.32 ± 0.23 ^a^
		Moderate stress	12.29 ± 0.13 ^b^	4.73 ± 0.23 ^a^	27.36 ± 0.23 ^a^
		Severe stress	10.67 ± 0.23 ^c^	2.67 ± 0.42 ^b^	18.08 ± 0.11 ^b^
		Two-Way ANOVA	*** *F*(I) = 113.483	*** *F*(I) = 78.233	* *F*(I) = 6.036
			*** *F*(R) = 105.897	*** *F*(R) = 44.860	*** *F*(R) = 25.857
			NS*F*(IxR) = 1.303	*** *F*(IxR) = 13.000	NS*F*(IxR) = 0.143
*Pegau harmala*	Non inoculated	Control	99.10 ± 0.32 ^a^	4.80 ± 0.69 ^a^	31.33 ± 0.11 ^a^
		Moderate stress	94.51 ± 0.61 ^b^	3.73 ± 0.23 ^b^	32.02 ± 0.20 ^a^
		Severe stress	88.57 ± 0.14 ^c^	2.40 ± 0.40 ^c^	14.73 ± 0.23 ^b^
	Inoculated	Control	106.21 ± 0.72 ^a^	5.27 ± 0.30 ^a^	46.75 ± 0.30 ^a^
		Moderate stress	105.03 ± 0.11 ^b^	4.47 ± 0.11 ^b^	40.08 ± 0.34 ^b^
		Severe stress	103.24 ± 0.30 ^c^	3.67 ± 0.11 ^c^	32.03 ± 0.20 ^c^
		Two-Way ANOVA	*** *F*(I) = 212.253	*** *F*(I) = 22.443	*** *F*(I) = 137.815
			*** *F*(R) = 29.521	*** *F*(R) = 44.328	*** *F*(R) = 69.148
			*** *F*(IxR) = 9.23	*** *F*(IxR) = 1.836	* *F*(IxR) = 6.037

Data are shown as means ± standard errors (n = 3). A two-way ANOVA was performed to determine the influence of microbial inoculation (I) and irrigation regimes (R) on the numbers of bacteria, fungi, and actinomycetes. The results are shown with the test statistic for each case (I: microbial inoculation; R: irrigation regimes; I x R: microbial inoculation x irrigation regimes) and as NS (non-significant) at the level *p* > 0.05; * significant at the level *p* < 0.05; *** significant at the level *p* < 0.001, respectively. One-way ANOVA was performed to determine the influence of irrigation on the numbers of bacteria, fungi, and actinomycetes for each inoculation treatment at the end of the experiment. Means for the same inoculation treatment showing different letters are significantly different from each other (*p* < 0.05) according to Duncan’s test.

**Table 3:** Physicochemical properties of non-amended and amended mine tailings, topsoil, and organo-mineral amendments (marble sludge and sheep manure).

Parameters	Mine Tailings	Topsoil	Marble Sludge	Sheep Manure	Amended Mine Tailings
pH H_2_O	2.36 ± 0.01	8.02 ± 0.07	10.33 ± 0.24	8.36 ± 0.11	7.55 ± 0.2
pH KCl	2.19 ± 0.12	7.88 ± 0.13	8.89 ± 0.11	7.92 ± 0.21	6.53 ± 0.16
EC (mS cm^−1^)	7.86	2.44	0.198	11.47	4.71 ± 0.04
Organic Carbon (%)	Nd *	1.13 ± 0.08	1.2 ± 0.05	40 ± 0.11	3.11 ± 0.05
Total N (%)	Nd *	0.17 ± 0.06	0.14 ± 0.16	1.28 ± 0.05	0.32 ± 0.01
Olsen-P (mg kg^−1^)	Nd *	43.42 ± 0.04	20 ± 1.23	1047 ± 11.24	42.84 ± 0.61
CaCO_3_ (%)	-	16.89 ± 0.14	57.42 ± 0.25	-	-
Texture	Loam	Sandy Loam	-	-	Sandy Loam
Clay (%)	7.47	5.58	-	-	6
Silt (%)	37.37	39.08	-	-	41
Sand (%)	50.47	52.89	-	-	53
Moisture content (%)	41.92	40.72	40.62	40.49	39.11
Total Cd (mg kg^−1^)	1.07 ± 0.04	0.08 ± 0.03	0.05 ± 0.01	0.12 ± 0.01	0.01 ± 0.001
Total Pb (mg kg^−1^)	178.48 ± 7.52	31.01 ± 7.37	1.2 ± 0.23	17.25 ± 0.41	45.47 ± 5.07
Total Zn (mg kg^−1^)	275.93 ± 10.66	102.47 ± 10.67	7.5 ± 0.31	73.82 ± 0.33	118.09 ± 7.53
Total Cu (mg kg^−1^)	1084.19 ± 64.2	424.08 ± 44.37	8.53 ± 0.22	17.03 ± 0.37	414.36 ± 43.32

Nd *: Not detected.

## Data Availability

All data generated or analyzed during this study are included in this published article. Further information is available from the corresponding authors upon reasonable request.

## References

[1] Franzaring J., Ancora S., Paoli L., Fongoh A., Büttner P., Fangmeier A., Schlosser S., Monaci F. (2018). Phytotoxicity of polymetallic mine wastes from southern Tuscany and Saxony. Ecotoxicol. Environ. Saf..

[2] Akcil A., Erust C., Ozdemiroglu S., Fonti V., Beolchini F. (2015). A review of approaches and techniques used in aquatic contaminated sediments: Metal removal and stabilization by chemical and biotechnological processes. J. Clean. Prod..

[3] Shackira A.M., Puthur J.T. (2019). Phytostabilization of heavy metals: Understanding of principles and practices. Plant-Metal Interactions.

[4] Tang C., Chen Y., Zhang Q., Li J., Zhang F., Liu Z. (2019). Effects of peat on plant growth and lead and zinc phytostabilization from lead-zinc mine tailing in southern China: Screening plant species resisting and accumulating metals. Ecotoxicol. Environ. Saf..

[5] Testiati E., Parinet J., Massiani C., Laffont-Schwob I., Rabier J., Pfeifer H.-R., Lenoble V., Masotti V., Prudent P. (2013). Trace metal and metalloid contamination levels in soils and in two native plant species of a former industrial site: Evaluation of the phytostabilization potential. J. Hazard. Mater..

[6] Dary M., Chamber-Pérez M., Palomares A., Pajuelo E. (2010). “In Situ” phytostabilisation of heavy metal polluted soils using *Lupinus luteus* inoculated with metal resistant plant growth promoting rhizobacteria. J. Hazard. Mater..

[7] Clemente R., Walker D.J., Pardo T., Martínez-Fernández D., Bernal M.P. (2012). The use of a halophytic plant species and organic amendments for the remediation of a trace elements-contaminated soil under semi-arid conditions. J. Hazard. Mater..

[8] Ma Y., Rajkumar M., Zhang C., Freitas H. (2016). Inoculation of *Brassica oxyrrhina* with plant growth promoting bacteria for the improvement of heavy metal phytoremediation under drought conditions. J. Hazard. Mater..

[9] Visser A., Kroes J., van Vliet M.T., Blenkinsop S., Fowler H.J., Broers H.P. (2012). Climate change impacts on the leaching of a heavy metal contamination in a small lowland catchment. J. Contam. Hydrol..

[10] Jha S., Srivastava R. (2018). Impact of drought on vegetation carbon storage in arid and semi-arid regions. Remote Sens. Appl. Soci. Environ..

[11] Parraga-Aguado I., González-Alcaraz M.N., Álvarez-Rogel J., Conesa H.M. (2014). Assessment of the employment of halophyte plant species for the phytomanagement of mine tailings in semiarid areas. Ecol. Eng..

[12] Boularbah A., Schwartz C., Bitton G., Morel J.L. (2006). Heavy metal contamination from mining sites in South Morocco: 2. Assessment of metal accumulation and toxicity in plants. Chemosphere.

[13] Boularbah A., Schwartz C., Bitton G., Morel J.L. (2006). Heavy metal contamination from mining sites in South Morocco: 1. Use of a biotest to assess metal toxicity of tailings and soils. Chemosphere.

[14] Mendez M.O., Maier R.M. (2008). Phytostabilization of mine tailings in arid and semiarid environments–an emerging remediation technology. Environ. Health Perspect..

[15] Maestri E., Marmiroli M., Visioli G., Marmiroli N. (2010). Metal tolerance and hyperaccumulation: Costs and trade-offs between traits and environment. Environ. Exp. Biol..

[16] Chaabani S., Abdelmalek-Babbou C., Ben Ahmed H., Chaabani A., Sebei A. (2012). Lead accumulation and phytostabilization potential of dominant plant species growing in a lead–zinc mine tailing. Environ. Earth Sci..

[17] Ali H., Khan E., Sajad M.A. (2013). Phytoremediation of heavy metals: Concepts and applications. Chemosphere.

[18] Meeinkuirt W., Pokethitiyook P., Kruatrachue M., Tanhan P., Chaiyarat R. (2012). Phytostabilization of a Pb-contaminated mine tailing by various tree species in potand field trial experiments. Int. J. Phytoremediation.

[19] Wasilkowski D., Nowak A., Płaza G., Mrozik A. (2017). Effects of pulp and Na bentonite amendments on the mobility of trace elements, soil enzymes activity and microbial parameters under ex situ aided phytostabilization. PLoS ONE.

[20] Zhou H., Chen C., Wang D., Arthur E., Zhang Z., Guo Z., Peng X., Mooney S.J. (2020). Mooney. Effect of long-term organic amendments on the full-range soil water retention characteristics of a Vertisol. Soil Tillage Res..

[21] Kang S.-W., Ahn K.-H. (2022). The Influence of Organic Matter Origin on the Chlorine Bulk Decay Coefficient in Reclaimed Water. Water.

[22] Taban M., Naeini S.A.R.M. (2006). Movahedi Naeini. Effect of aquasorb and organic compost amendments on soil water retention and evaporation with different evaporation potentials and soil textures. Commun. Soil Sci. Plant Anal..

[23] Ju W., Liu L., Fang L., Cui Y., Duan C., Wu H. (2019). Impact of co-inoculation with plant-growth-promoting rhizobacteria and *rhizobium* on the biochemical responses of alfalfa-soil system in copper contaminated soil. Ecotoxicol. Environ. Saf..

[24] Zainab N., Din B.U., Javed M.T., Afridi M.S., Mukhtar T., Kamran M.A., Chaudhary H.J. (2020). Deciphering metal toxicity responses of flax (*Linum usitatissimum* L.) with exopolysaccharide and ACC-deaminase producing bacteria in industrially contaminated soils. Plant Physiol. Biochem..

[25] Benidire L., Pereira S., Aboudrar W., Hafidi M., Castro P., Boularbah A. (2022). Remediation of metal-contaminated mine tailings by the application of organic and mineral amendments. J. Soils Sediments.

[26] Rani R., Kumar V., Gupta P., Chandra A. (2021). Potential use of *Solanum lycopersicum* and plant growth promoting rhizobacterial (PGPR) strains for the phytoremediation of endosulfan stressed soil. Chemosphere.

[27] Madline A., Benidire L., Boularbah A. (2021). Alleviation of salinity and metal stress using plant growth-promoting rhizobacteria isolated from semiarid Moroccan copper-mine soils. Environ. Sci. Pollut. Res..

[28] Sandhya V.S.K.Z., Ali S.Z., Grover M., Reddy G., Venkateswarlu B. (2020). Effect of plant growth promoting *Pseudomonas* spp. on compatible solutes, antioxidant status and plant growth of maize under drought stress. Plant Growth Regul..

[29] Wang X., Tang C., Severi J., Butterly C.R., Baldock J.A. (2016). Rhizosphere priming effect on soil organic carbon decomposition under plant species differing in soil acidification and root exudation. New Phytol..

[30] Gispert M., Emran M., Pardini G., Doni S., Ceccanti B. (2013). The impact of land management and abandonment on soil enzymatic activity, glomalin content and aggregate stability. Geoderma.

[31] Yadav B.K., Akhtar M., Panwar J. (2015). Rhizospheric plant-microbe interactions: Key factors to soil fertility and plant nutrition. Plant Microbes Symbiosis: Applied Facets.

[32] Bowsher A.W., Ali R., Harding S.A., Tsai C.J., Donovan L.A. (2016). Evolutionary divergences in root exudate composition among ecologically-contrasting Helianthus species. PLoS ONE.

[33] Gamage SS W., Masakorala K., Brown M.T., Gamage S.M.K.W. (2021). Comparative phytoremediation potentials of *Impatiens balsamina* L. and *Crotalaria retusa* L. for soil contaminated with used lubricating oil. Environ. Adv..

[34] Chaparro J.M., Sheflin A.M., Manter D.K., Vivanco J.M. (2012). Manipulating the soil microbiome to increase soil health and plant fertility. Biol. Fertil. Soils.

[35] Kim J.H., Kim S.-J., Nam I.-H. (2021). Effect of treating acid sulfate soils with phosphate solubilizing bacteria on germination and growth of tomato (*Lycopersicon esculentum* L.). Int. J. Environ. Res. Public Health.

[36] Altaf M. (2021). Functional diversity of nitrogen-fixing plant growth-promoting Rhizobacteria: The story so far. Soil Nitrogen Ecology.

[37] Zeng Q., Ding X., Wang J., Han X., Iqbal H., Bilal M. (2022). Insight into soil nitrogen and phosphorus availability and agricultural sustainability by plant growth-promoting rhizobacteria. Environ. Sci. Pollut. Res..

[38] Pramanik K., Mitra S., Sarkar A., Maiti T.K. (2018). Alleviation of phytotoxic effects of cadmium on rice seedlings by cadmium resistant PGPR strain Enterobacter aerogenes MCC 3092. J. Hazard. Mater..

[39] Benidire L., Madline A., Pereira S.I.A., Castro P.M.L., Boularbah A. (2020). Synergistic effect of organo-mineral amendments and plant growth-promoting rhizobacteria (PGPR) on the establishment of vegetation cover and amelioration of mine tailings. Chemosphere.

[40] Daraz U., Li Y., Sun Q., Zhang M., Ahmad I. (2021). Inoculation of Bacillus spp. modulate the soil bacterial communities and available nutrients in the rhizosphere of vetiver plant irrigated with acid mine drainage. Chemosphere.

[41] Madejón P., Navarro-Fernández C.M., Madejón E., López-García Á., Marañón T. (2021). Plant response to mycorrhizal inoculation and amendments on a contaminated soil. Sci. Total Environ..

[42] Tirry N., Kouchou A., Laghmari G., Lemjereb M., Hnadi H., Amrani K., El Ghachtouli N. (2021). Improved salinity tolerance of *Medicago sativa* and soil enzyme activities by PGPR. Biocatal. Agric. Biotechnol..

[43] Dey G., Banerjee P., Maity J.P., Sharma R.K., Gnanachandrasamy G., Huang Y.H., Chen C.Y. (2022). Heavy metals distribution and ecological risk assessment including arsenic resistant PGPR in tidal mangrove ecosystem. Mar. Pollut. Bull..

[44] Sardans J., Peñuelas J. (2005). Drought decreases soil enzyme activity in a Mediterranean *Quercus ilex* L. forest. Soil. Biol. Biochem..

[45] Pohlon E., Fandino A.O., Marxsen J. (2013). Bacterial community composition and extracellular enzyme activity in temperate streambed sediment during drying and rewetting. PLoS ONE.

[46] Siebielec S., Siebielec G., Klimkowicz-Pawlas A., Gałązka A., Grządziel J., Stuczyński T. (2020). Impact of water stress on microbial community and activity in sandy and loamy soils. Agronomy.

[47] Schreckinger J., Mutz M., Mendoza-Lera C., Frossard A. (2021). Attributes of drying define the structure and functioning of microbial communities in temperate riverbed sediment. Front. Microbiol..

[48] Sardans J., Peñuelas J. (2010). Soil enzyme activity in a Mediterranean forest after six years of drought. Soil. Sci. Soc. Am. J..

[49] Gao W., Reed S.C., Munson S.M., Rui Y., Fan W., Zheng Z., Hao Y. (2021). Responses of soil extracellular enzyme activities and bacterial community composition to seasonal stages of drought in a semiarid grassland. Geoderma.

[50] Chaudhary D.R., Rathore A.P., Sharma S. (2022). Effect of halotolerant plant growth promoting rhizobacteria inoculation on soil microbial community structure and nutrients. Appl. Soil Ecol..

[51] Zhu L., Wang X., Chen F., Li C., Wu L. (2019). Effects of the successive planting of *Eucalyptus urophylla* on soil bacterial and fungal community structure, diversity, microbial biomass, and enzyme activity. Land Degrad. Dev..

[52] Lin H., Liu C., Li B., Dong Y. (2021). *Trifolium repens* L. regulated phytoremediation of heavy metal contaminated soil by promoting soil enzyme activities and beneficial rhizosphere associated microorganisms. J. Hazard. Mater..

[53] Piromyou P., Noisangiam R., Uchiyama H., Tittabutr P., Boonkerd N., Teaumroong N. (2013). Indigenous microbial community structure in rhizosphere of Chinese kale as affected by plant growth-promoting rhizobacteria inoculation. Pedosphere.

[54] Ilyas N., Mumtaz K., Akhtar N., Yasmin H., Sayyed R.Z., Khan W., Ali Z. (2020). Exopolysaccharides producing bacteria for the amelioration of drought stress in wheat. Sustainability.

[55] Kumar A., Singh S., Gaurav A.K., Srivastava S., Verma J.P. (2020). Plant growth-promoting bacteria: Biological tools for the mitigation of salinity stress in plants. Front. Microbiol..

[56] Shi G., Xia S., Ye J., Huang Y., Liu C., Zhang Z. (2015). PEG-simulated drought stress decreases cadmium accumulation in *castor bean* by altering root morphology. Environ. Exp. Bot..

[57] Wang J.Q., Li H., Liu Q., Xiang D. (2019). Effects of drought stress on root development and physiological characteristics of sweet potato at seedling stage. J. Appl. Ecol..

[58] Xiao X.X. (2002). The physiological and biochemical response of longan (Dimocarpus longan Lour.) to aluminum stress and rectification of aluminum toxicity. Fujian J. Agric. Sci..

[59] Orrego F., Ortíz-Calderón C., Lutts S., Ginocchio R. (2020). Effect of single and combined Cu, NaCl and water stresses on three *Atriplex* species with phytostabilization potential. S. Afr. J. Bot..

[60] Nedjimi B., Beladel B., Guit B. (2012). Biodiversity of halophytic vegetation in chott *Zehrez* lake of Djelfa (Algeria). Am. J. Plant Sci..

[61] Geng M., Xu M., Xiao H., Wang H., He L., Zhao Z., Yu M. (2012). Protective role of mucilage against Al toxicity to root apex of pea (*Pisum sativum*). Acta Physiol. Plant..

[62] Liao M., Xie X.M. (2004). Cadmium release in contaminated soils due to organic acids. Pedosphere.

[63] Bali A.S., Sidhu GP S., Kumar V. (2020). Root exudates ameliorate cadmium tolerance in plants: A review. Environ. Chem. Lett..

[64] Yamaguchi M., Sharp R.E. (2010). Complexity and coordination of root growth at low water potentials: Recent advances from transcriptomic and proteomic analyses. Plant Cell Environ..

[65] Naseem H., Ahsan M., Shahid M.A., Khan N. (2018). Exopolysaccharides producing rhizobacteria and their role in plant growth and drought tolerance. J. Basic Microbiol..

[66] Abdelkrim S., Jebara S.H., Saadani O., Abid G., Taamalli W., Zemni H., Jebara M. (2020). In Situ effects of Lathyrus sativus-PGPR to remediate and restore quality and fertility of Pb and Cd polluted soils. Ecotoxicol. Environ. Saf..

[67] Morcillo R.J., Manzanera M. (2021). The effects of plant-associated bacterial exopolysaccharides on plant abiotic stress tolerance. Metabolites.

[68] Ahmed B., Shahid M., Syed A., Rajput V.D., Elgorban A.M., Minkina T., Lee J. (2021). Drought tolerant *Enterobacter* sp./*Leclercia adecarboxylata* secretes indole-3-acetic acid and other biomolecules and enhances the biological attributes of *Vigna radiata* (L.) R. Wilczek in water deficit conditions. Biology.

[69] Hou X., Teng W., Hu Y., Yang Z., Li C., Scullion J., Zheng R. (2020). Potential phytoremediation of soil cadmium and zinc by diverse ornamental and energy grasses. BioResources.

[70] Benidire L., Pereira S.I.A., Castro P.M.L., Boularbah A. (2016). Assessment of plant growth-promoting bacterial populations in the rhizosphere of metallophytes from the Kettara mine, Marrakech. Environ. Sci. Pollut. Res..

[71] El Hamiani O., El Khalil H., Sirguey C., Ouhammou A., Bitton G., Schwartz C., Boularbah A. (2015). Metal concentrations in plants from mining areas in South Morocco: Health risks assessment of consumption of edible and aromatic plants. CLEAN–Soil. Air Water.

[72] Olsen S.R., Sommers L.E., Page A.L., Miller R.H., Keeney D.R. (1982). Phosphorus. Methods of Soil Analysis Part 2.

[73] Aubert G. (1978). Methodes D’analyses Des Sols.

[74] Tabatabai M.A., Bremner J.M. (1969). Use of p-nitrophenyl phosphate for assay of soil phosphatase activity. Soil. Biol. Biochem..

[75] Alef K., Nannipieri P. (1995). Methods in Applied Soil Microbiology and Biochemistry.

[76] Kandeler E., Gerber H. (1988). Short-term assay of soil urease activity using colorimetric determination of ammonium. Biol. Fertil. Soils.

